# Ferroptosis: key regulatory pathways and their implications in cardiovascular pathophysiology

**DOI:** 10.3389/fcvm.2026.1732010

**Published:** 2026-04-15

**Authors:** Kaishen Cao, Xiaoping Peng, Xiang Wang

**Affiliations:** 1 Department of Cardiology, The First Affiliated Hospital, Jiangxi Medical College, Nanchang University, Nanchang, China; 2 Jiangxi Medical College, Nanchang University, Nanchang, China; 3 Jiangxi Hypertension Research Institute, Nanchang, China; 4 Jiangxi Key Laboratory of Neurological Diseases, Department of Cardiology, The First Affiliated Hospital, Jiangxi Medical College, Nanchang University, Nanchang, China; 5 Academician Workstation of Cardiovascular Innovative Materials, Nanchang, China

**Keywords:** cardiovascular diseases, ferroptosis, iron metabolism, lipid peroxidation, spatial metabolomics

## Abstract

Cardiovascular diseases (CVDs) remain a leading global health burden, necessitating novel insights into their pathogenesis and therapeutic strategies. Ferroptosis, an iron-dependent form of regulated cell death driven by lipid peroxidation, has emerged as a pivotal mechanism in CVD progression. This review comprehensively synthesizes current knowledge on the molecular drivers of ferroptosis, including dysregulated iron metabolism, glutathione peroxidase 4 (GPX4) inactivation, and redox imbalance orchestrated by Nrf2, AMPK, and p53. Subcellular organelles such as mitochondria, lysosomes, and the endoplasmic reticulum are highlighted as critical hubs for initiating or amplifying ferroptotic signals through oxidative stress, metabolic dysfunction, and organelle-specific interactions. The role of ferroptosis in major cardiovascular pathologies—atherosclerosis, vascular calcification, heart failure, ischemia-reperfusion injury, and arrhythmias—is systematically explored, emphasizing its contribution to cellular damage, inflammation, and tissue remodeling. Notably, this review incorporates discussions on spatial metabolomics as a powerful analytical tool, highlighting its unique capacity to decipher region-specific metabolic alterations and spatial distribution patterns of key molecules involved in ferroptosis, thereby providing deeper insights into the spatiotemporal dynamics of ferroptotic mechanisms in CVDs. Furthermore, emerging therapeutic strategies targeting ferroptosis, including iron chelators, lipid peroxidation inhibitors, and metabolic modulators (e.g., metformin, trimetazidine), are discussed for their potential to mitigate cardiovascular damage. By bridging molecular mechanisms (enhanced by spatial metabolomics insights) to clinical applications, this review underscores ferroptosis as a promising therapeutic target, advocating for further research to translate these insights into precision interventions for CVD management.

## Introduction

Cardiovascular diseases (CVDs) remain a major global health threat. According to data from the World Health Organization (WHO), approximately 19.8 million people worldwide succumbed to CVDs in 2022 (1)—a staggering statistic that underscores the profound and far-reaching impact of these diseases on global public health. In China, the 2023 Report on Cardiovascular Health and Diseases further highlights the severity of the issue: an estimated 330 million individuals are affected by some form of CVD, and these conditions account for nearly two-fifths of all deaths in the country. Collectively, these figures emphasize the urgent need for innovative research to unravel the underlying pathogenesis of CVDs and develop effective therapeutic interventions, with the ultimate goal of improving patient outcomes.

In recent years, the scientific community has made remarkable strides in elucidating the mechanisms of cell death and their implications for disease pathogenesis. Among the most pivotal discoveries is ferroptosis—a newly identified form of programmed cell death that is fundamentally distinct from well-characterized pathways such as apoptosis, necrosis, and autophagy (2, 3). Ferroptosis is defined by two core features: its strict dependence on iron and its close association with lipid peroxidation, specifically the oxidative damage to polyunsaturated fatty acids (PUFAs) in cellular membranes. This progressive oxidative process ultimately disrupts membrane integrity and triggers cell death. Critically, ferroptosis is driven by the depletion or functional inactivation of glutathione peroxidase 4 (GPX4)—a key enzyme in the cellular antioxidant defense system that normally suppresses lipid peroxidation (4, 5). Unlike traditional cell death mechanisms, ferroptosis can be specifically modulated by interventions targeting lipid peroxidation (e.g., lipophilic antioxidants) or iron metabolism (e.g., iron chelators) (3, 6).

In recent years, the role of ferroptosis in CVDs has garnered substantial attention from researchers, as it holds the potential to provide novel perspectives for understanding disease progression and developing targeted treatments. While accumulating evidence has established a link between ferroptosis and various cardiovascular pathologies, the precise molecular mechanisms governing its involvement in CVD progression remain incompletely elucidated. Against this backdrop, this review aims to: (1) explore the emerging role of ferroptosis in the cardiovascular system, (2) comprehensively summarize current research findings on ferroptosis-related mechanisms in CVDs, and (3) investigate its potential as a therapeutic target. By addressing these objectives, this review seeks to offer new strategies for the clinical management of CVDs.

## Molecular and metabolic drivers of ferroptosis

Rather than being driven by a single molecular pathway, ferroptosis emerges from the coordinated dysregulation of iron metabolism, lipid peroxidation, and cellular antioxidant systems. Increasing evidence suggests that these processes are not uniformly regulated at the cellular level but are highly dependent on subcellular compartmentalization and metabolic context, which adds an additional layer of complexity beyond classical models.

### Iron metabolism and spatial iron redistribution

**Figure 1:**
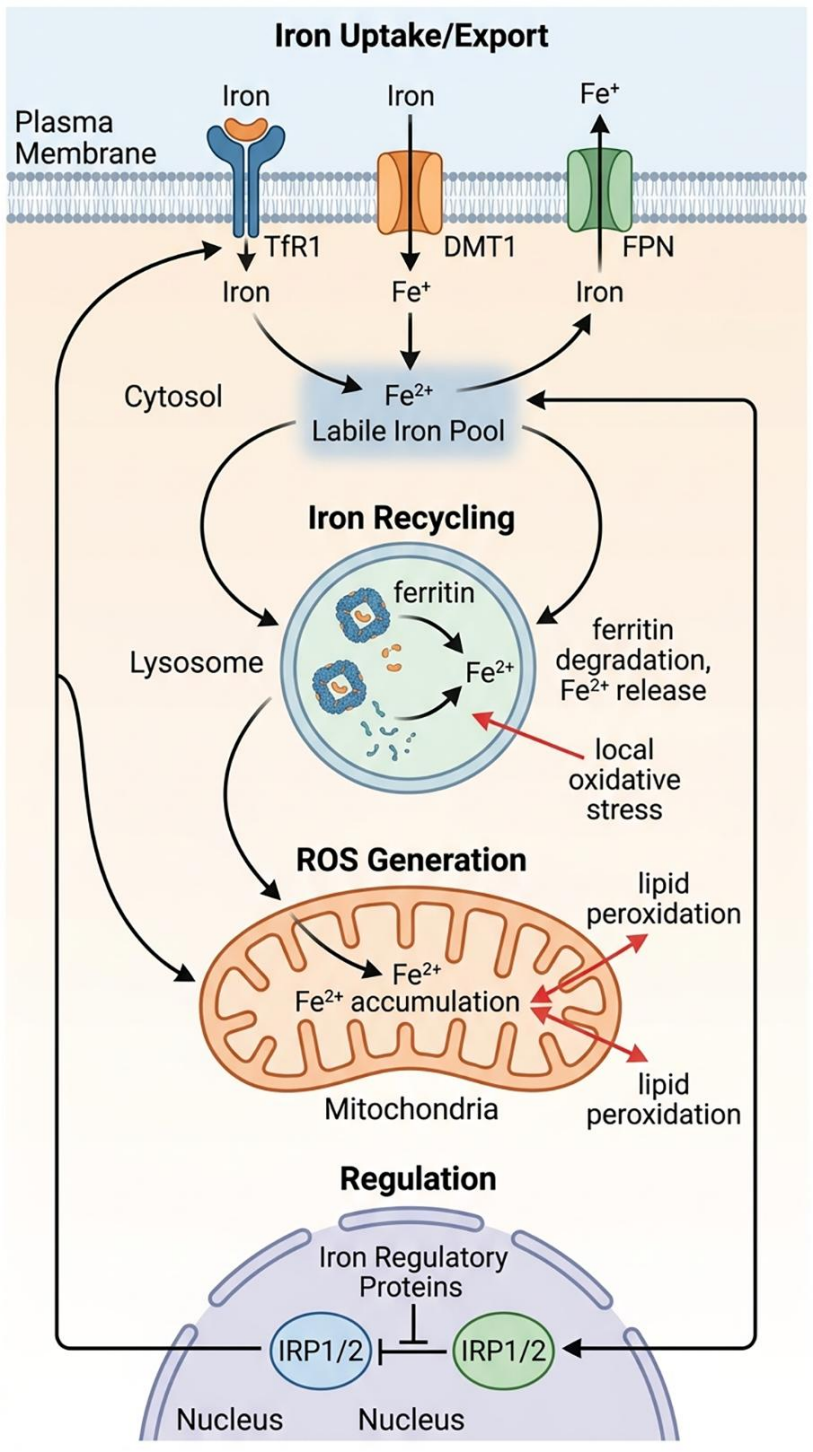
Iron metabolism and spatial iron redistribution. Cellular iron homeostasis is maintained via coordinated uptake through TfR1 and DMT1, ferritin sequestration and lysosomal recycling, and efflux via ferroportin (FPN), collectively regulating the labile iron pool. Spatial partitioning of labile iron into mitochondria drives ROS production and lipid peroxidation, processes post-transcriptionally controlled by iron regulatory proteins (IRP1/2) to link subcellular iron distribution to ferroptotic signaling. TfR1,transferrin receptor 1; DMT1, divalent metal transporter 1; FPN, ferroportin; IRP1, Iron Regulatory Protein 1.

Iron metabolism is tightly controlled through coordinated regulation of uptake, storage, and export, involving key components such as TfR1, DMT1, ferritin, and ferroportin (6–10). Post-transcriptional regulation by IRP1/2 further ensures dynamic adaptation to intracellular iron levels (11–13). While disruption of this network has long been considered a prerequisite for ferroptosis, recent studies suggest that total cellular iron levels alone are insufficient to explain ferroptotic sensitivity (Figure 1).

Instead, emerging evidence highlights the importance of labile iron redistribution within specific organelles. For example, mitochondrial Fe²+ accumulation has been shown to directly enhance ROS generation and lipid peroxidation (14), whereas lysosomal dysfunction can impair ferritin degradation and alter iron recycling, thereby increasing local oxidative stress (15). These findings indicate that ferroptosis may be triggered by localized iron overload rather than global iron excess.

However, key questions remain unresolved. It is still unclear how iron trafficking between organelles is coordinated under stress conditions, and whether specific iron pools act as dominant drivers of ferroptosis in different disease contexts. Addressing these issues will be critical for understanding ferroptosis at a systems level.

### Lipid peroxidation as a spatially regulated process

Lipid peroxidation is the defining biochemical feature of ferroptosis, involving the oxidation of PUFA-containing phospholipids mediated by enzymes such as LOXs and regulated by ACSL4 (16–18). ROS generated via iron-dependent reactions initiate this process, leading to progressive membrane damage and cell death (19). Traditionally, GPX4 has been viewed as the central suppressor of ferroptosis by detoxifying lipid peroxides (20, 21). However, recent findings challenge this simplified model. First, lipid peroxidation appears to occur in a compartment-specific manner, with certain membrane domains (e.g., mitochondrial or ER-associated membranes) being more susceptible. Second, ferroptotic cell death can still proceed under conditions where GPX4 is partially active, suggesting the existence of GPX4-independent lipid detoxification failure mechanisms. These observations raise important questions regarding how lipid composition, membrane architecture, and local redox environments interact to determine ferroptosis sensitivity. In particular, the relative contributions of enzymatic vs. non-enzymatic lipid peroxidation pathways remain incompletely defined.

### Antioxidant and metabolic signaling

**Figure 2:**
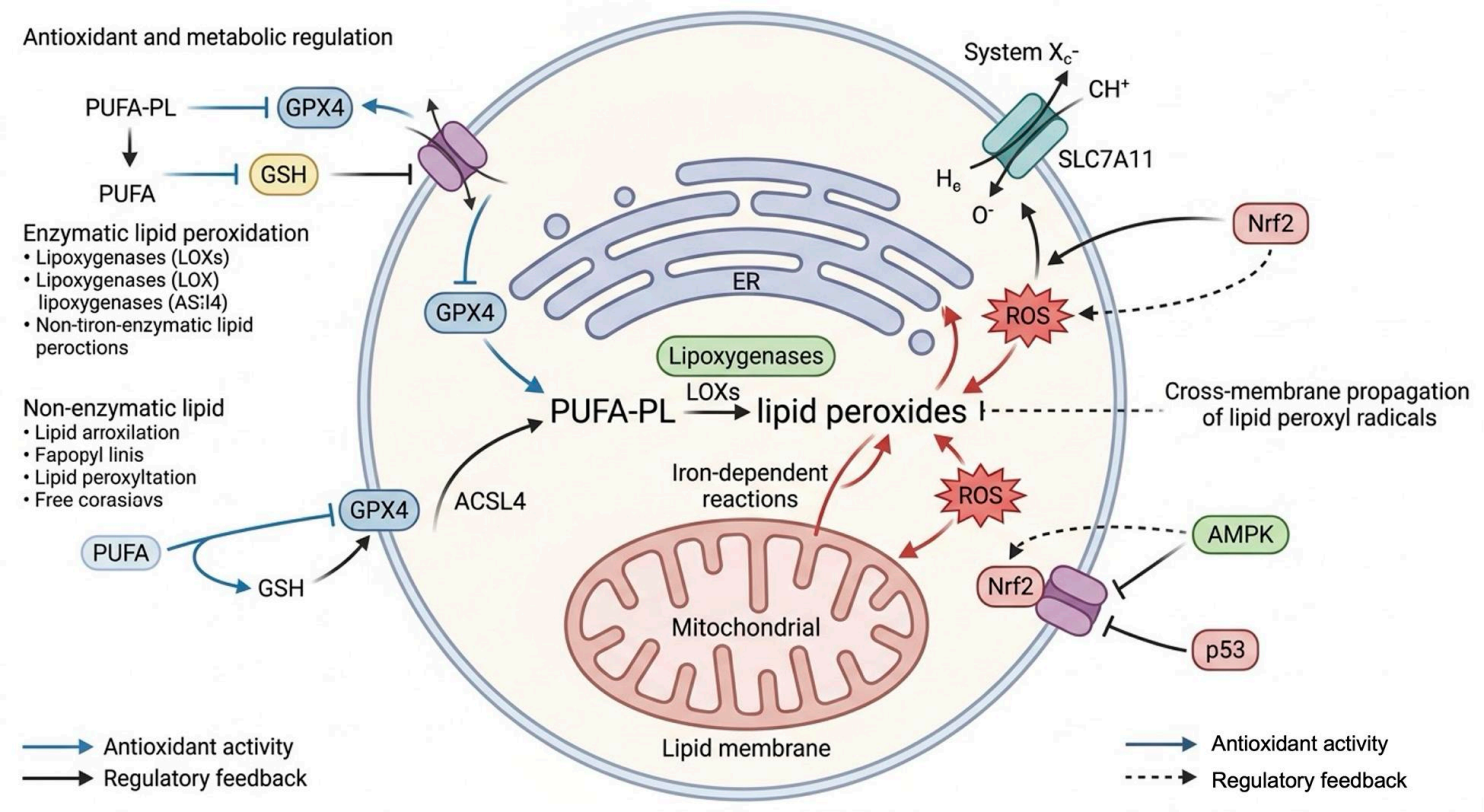
Antioxidant and metabolic signaling. Coordinated antioxidant and metabolic pathways govern lipid peroxidation, where PUFA-PL are metabolized by ACSL4 and LOXs to generate lipid peroxides via iron-dependent reactions. GPX4, supported by GSH, neutralizes these peroxides, while the System Xc^−^ transporter (SLC7A11), Nrf2, AMPK, and p53 orchestrate redox and metabolic feedback to modulate ROS production and lipid peroxidation propagation across membranes. PUFA-PL, polyunsaturated fatty acid-containing phospholipids; ACSL4, acyl-CoA synthetase long-chain family member 4; LOXs, lipoxygenases; GPX4, Glutathione peroxidase 4; GSH, glutathione; Nrf2, nuclear factor erythroid 2-related factor 2; AMPK, AMP-activated protein kinase; ROS, reactive oxygen species.

Cellular resistance to ferroptosis is largely governed by antioxidant systems and metabolic signaling pathways. Nrf2 plays a central role by inducing the expression of genes involved in glutathione synthesis, GPX4 activity, and iron sequestration (22–25), while AMPK regulates lipid metabolism and iron export through modulation of ACC and ferroportin (26–28). Together, these pathways help maintain redox balance and limit lipid peroxidation (Figure 2).

However, their roles are not universally protective. Increasing evidence suggests that both Nrf2 and AMPK exhibit context-dependent and even opposing effects. For instance, sustained activation of Nrf2 has been linked to ferroptosis resistance in cancer cells, potentially contributing to therapy failure, whereas under severe metabolic stress, AMPK activation may promote ferroptosis by altering energy homeostasis.

Similarly, the cystine/glutamate antiporter System Xc^−^ and its subunit SLC7A11 are essential for maintaining glutathione levels (29, 30), yet their regulation is highly dynamic. The tumor suppressor p53 can repress SLC7A11 to promote ferroptosis, while simultaneously influencing iron metabolism through ferroportin and ferritin regulation (31, 32). Notably, different p53 mutations exhibit divergent effects on ferroptosis, further complicating this regulatory network.

These findings underscore that ferroptosis is not governed by linear pathways but by a highly dynamic and context-dependent network, in which the same regulator may exert distinct effects depending on cellular state, stress intensity, and disease context.

### Organelle-specific contributions to ferroptosis

The execution of ferroptosis depends not only on the activation of iron-dependent lipid peroxidation but also on how these reactions are spatially initiated and propagated between organelles. In this context, distinct subcellular compartments do not merely host ferroptotic processes but impose rate-limiting steps that determine whether lipid peroxidation remains contained or evolves into irreversible membrane damage.

Lysosomes provide a primary source of redox-active iron that seeds ferroptotic reactions. NCOA4-mediated ferritinophagy selectively increases the lysosomal Fe²+ pool (33), where the acidic microenvironment favors Fenton chemistry. Importantly, this creates a localized hotspot for hydroxyl radical generation that preferentially targets lysosomal membranes. Once lipid peroxidation is initiated at this site, lysosomal membrane permeabilization permits the release of iron into the cytosol (34), effectively converting a compartmentalized reaction into a cell-wide oxidative process. This transition represents a critical amplification step that lowers the threshold for ferroptosis.

The endoplasmic reticulum defines the susceptibility of cellular membranes to this iron-driven oxidative input. Through ACSL4- and LPCAT3-dependent phospholipid remodeling, the ER controls the enrichment of polyunsaturated fatty acyl chains within membrane phospholipids, thereby determining the density of peroxidation-sensitive substrates (35, 36). In parallel, PERK–eIF2*α*–ATF4 signaling rewires cystine uptake and glutathione synthesis, directly constraining GPX4-dependent detoxification capacity (37). As a result, ER stress does not simply accompany ferroptosis but shifts the balance between lipid peroxide generation and clearance, thereby dictating whether oxidative damage can be buffered.

Nuclear signaling establishes the permissive or restrictive state for these processes by coordinating transcriptional programs that control both iron handling and antioxidant defenses. p53-dependent repression of SLC7A11 limits cystine import and reduces glutathione availability (38), while Nrf2-driven transcription enhances ferritin expression and NADPH regeneration (39). The net ferroptotic outcome therefore depends on how these opposing programs are integrated over time. Under sustained stress, such as in cardiomyocytes exposed to toxic stimuli, this regulatory balance can collapse, leading to a coordinated increase in iron availability and a decrease in lipid peroxide detoxification capacity.

Peroxisomes contribute to the propagation phase of ferroptosis by modulating both reactive oxygen species flux and membrane composition. Peroxisomal *β*-oxidation generates hydrogen peroxide that can diffuse into adjacent compartments, reinforcing oxidative stress initiated elsewhere (40). At the same time, peroxisome-dependent synthesis of ether phospholipids alters membrane packing and may influence the accessibility of polyunsaturated lipids to peroxidation. This positions peroxisomes as regulators of how efficiently lipid peroxidation spreads across membrane systems (41).

The Golgi apparatus, although less directly linked to canonical ferroptosis pathways, may influence membrane repair and lipid redistribution following oxidative damage. Disruption of Golgi-dependent trafficking can impair the delivery of newly synthesized lipids and proteins required for membrane turnover, thereby limiting the capacity of cells to counteract ongoing lipid peroxidation (42, 43). In this way, Golgi dysfunction may not initiate ferroptosis but can determine its irreversibility.

**Figure 3:**
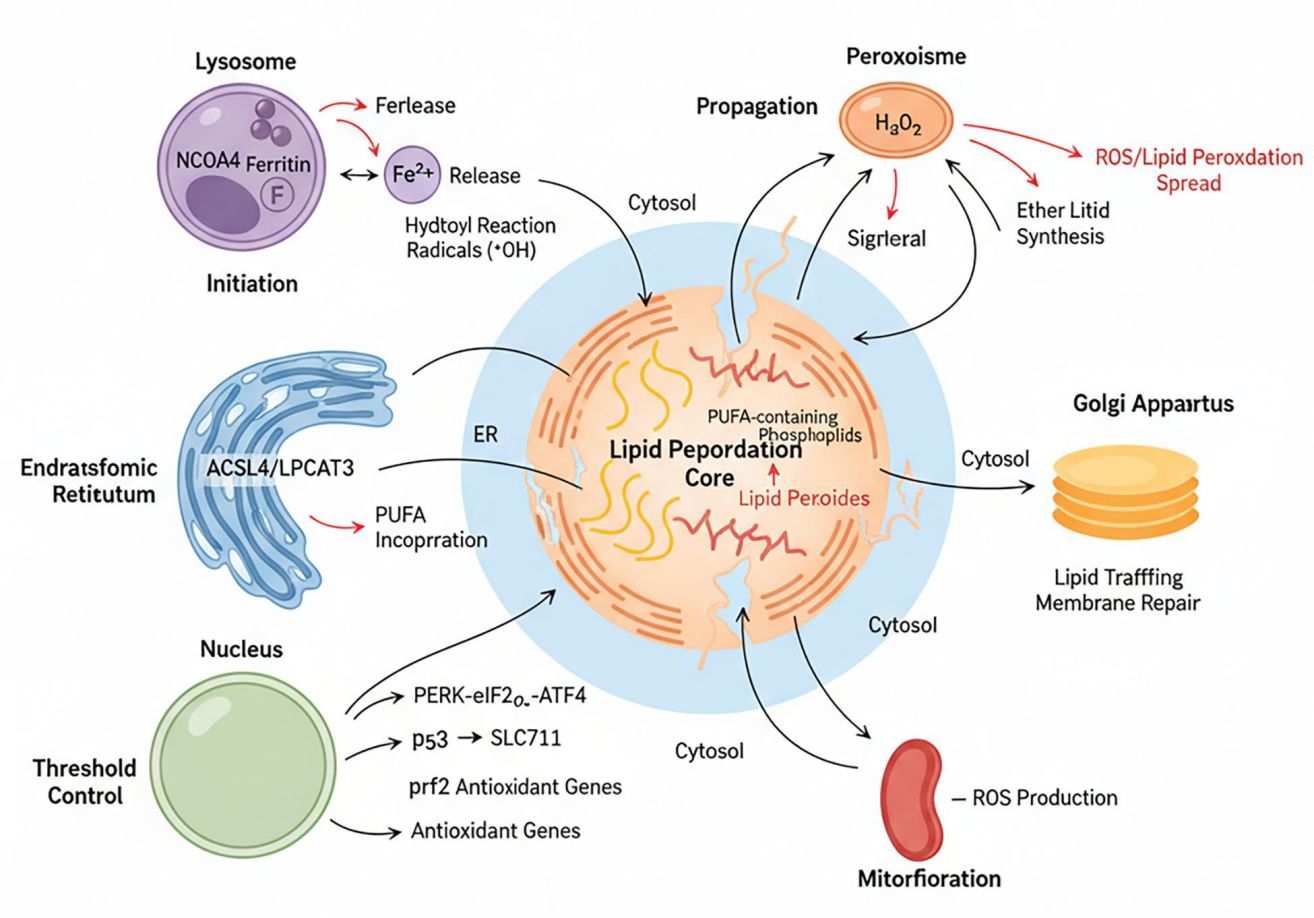
Organelle-Specific contributions to ferroptosis. Distinct subcellular organelles orchestrate sequential stages of ferroptosis: lysosomes initiate iron release from ferritin to drive hydroxyl radical formation, while the ER incorporates PUFAs into membrane phospholipids via ACSL4/LPCAT3 to prime lipid peroxidation. Peroxisomes propagate ROS and lipid peroxidation spread through ether lipid synthesis, mitochondria amplify ROS production, and the Golgi apparatus mediates lipid trafficking and membrane repair, with the nucleus governing threshold control via PERK-eIF2*α*-ATF4, p53-SLC7A11, and Nrf2-mediated antioxidant gene expression. ER, endoplasmic reticulum; PUFAs, polyunsaturated fatty acids.

Taken together, ferroptosis can be viewed as a spatially coupled process in which lysosomal iron release initiates localized oxidation, ER-dependent lipid remodeling defines substrate vulnerability, nuclear programs set the antioxidant threshold, and peroxisomes together with the Golgi apparatus regulate the propagation and resolution of membrane damage. This coordinated failure of compartmentalized control may be particularly relevant in cardiomyocytes, where tight organelle coupling facilitates rapid system-wide propagation of oxidative injury (Figure 3).

## Role of ferroptosis in cardiovascular diseases

**Figure 4:**
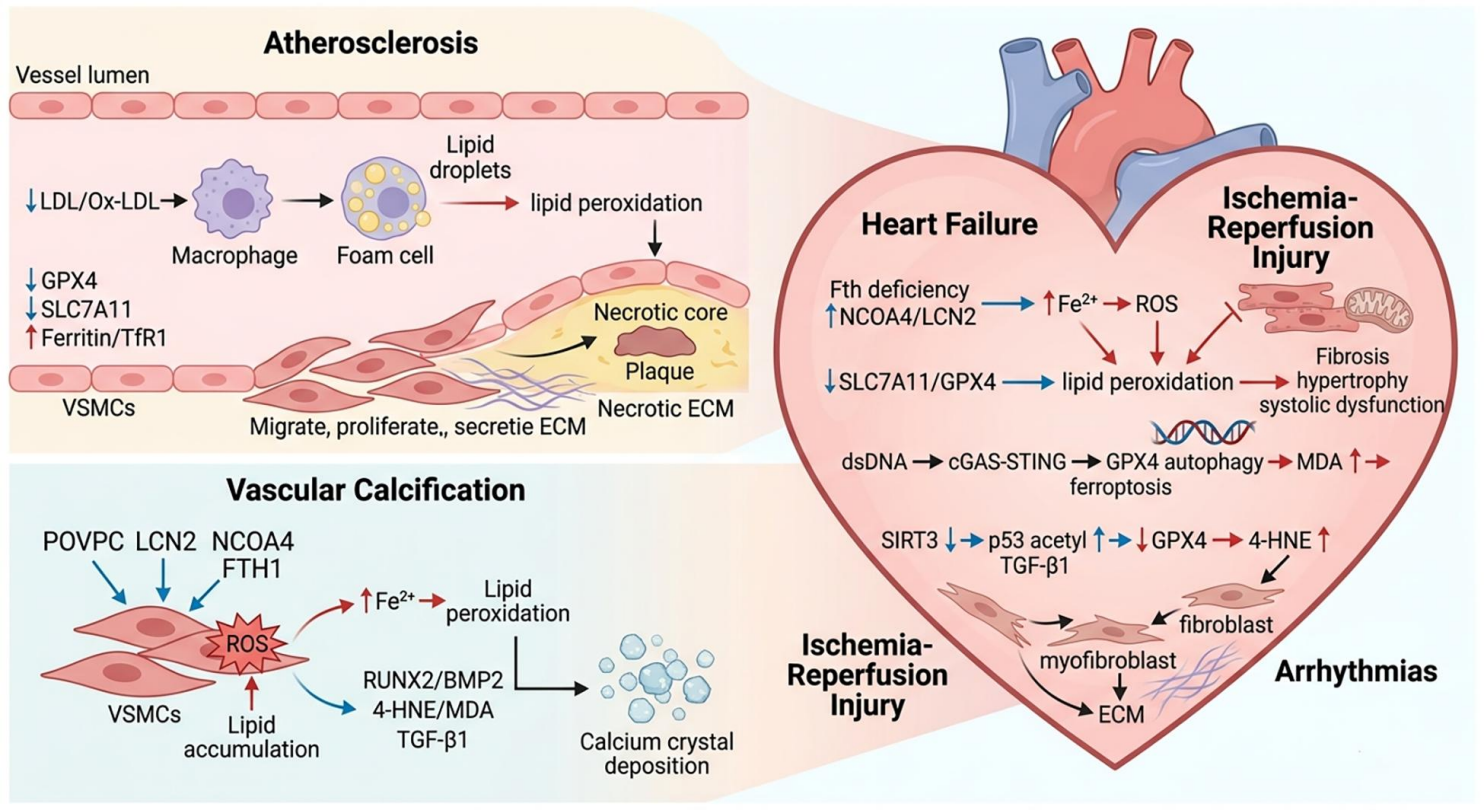
Role of ferroptosis in cardiovascular diseases.

Accumulating evidence indicates that dysregulated ferroptosis participates in the pathological processes of multiple cardiovascular disorders. Disturbances in iron metabolism, downregulation of key antioxidant systems, and mitochondrial dysfunction collectively drive ferroptotic cell death in various cardiac and vascular cell types. Given its broad contribution to cardiovascular injury, ferroptosis has emerged as a key molecular mechanism and potential therapeutic target in cardiovascular research (Figure 4, Table 1).

**Table 1:** Key ferroptotic mechanisms in Major cardiovascular diseases.

Cardiovascular Disease	Core Cell Types Involved	Key Regulatory Molecules/Pathways	Core Mechanisms
Atherosclerosis	Vascular endothelial cells, macrophages, VSMCs	GPX4, SLC7A11 (49, 50), TfR1 (51), Nrf2 (50), ox-LDL (45)	Ox-LDL induces GSH depletion and lipid peroxidation; ferroptosis of foam cells promotes plaque vulnerability and inflammation
Vascular Calcification	VSMCs	LCN2/NCOA4/FTH1 (55), SLC7A11/GPX4 (58), RUNX2, BMP2 (56)	LCN2-mediated ferritinophagy releases free Fe²+; ferroptosis drives VSMC osteogenic transdifferentiation and calcium phosphate deposition
Heart Failure	Cardiomyocytes	Fth (62), SLC7A11, GPX4 (61), DHODH (65), Nrf2 (63), LGR6-STAT3-Pgc1*α* (66)	Fth deficiency disrupts iron storage and redox balance; ferroptosis induces myocardial hypertrophy, fibrosis, and systolic dysfunction
Ischemia-Reperfusion Injury	Cardiomyocytes	cGAS-STING (67), GPX4 (67), Nrf2 (72), SLC7A11 (39), TfR2*β* (68), DMT1 (68)	Ischemia-induced cGAS-STING activation degrades GPX4; reperfusion triggers iron overload and lipid peroxidation
Arrhythmias (Atrial Fibrillation)	Cardiomyocytes, cardiac fibroblasts	SIRT3, p53 (74), GPX4 (75), TSNA-5008A (76)	SIRT3 deficiency enhances p53 acetylation and ferroptosis; lipid peroxides promote atrial fibrosis and arrhythmogenic substrate formation

VSMCs, vascular smooth muscle cells; GPX4, glutathione peroxidase 4; SLC7A11, Solute Carrier Family 7 Member 11; TfR1, transferrin receptor 1; Nrf2, Nuclear factor erythroid 2-related factor 2; ox-LDL, oxidized low-density lipoprotein; GSH, glutathione; NCOA4, nuclear receptor coactivator 4; FTH1, ferritin heavy chain 1; RUNX2, Runt-related transcription factor 2; BMP2, Bone morphogenetic protein 2; LGR6, Leucine-rich repeat-containing G-protein coupled receptor 6; STAT3, Signal transducer and activator of transcription 3; Pgc1α, Peroxisome proliferator-activated receptor gamma coactivator 1-α; cGAS, cyclic GMP-AMP synthase; STING, stimulator of interferon genes; DMT1, divalent metal transporter 1; SIRT3, sirtuin 3.

### Atherosclerosis

Atherosclerosis, a chronic inflammatory vascular disorder marked by intimal lipid deposition and persistent inflammation in large and medium-sized arteries, remains the leading global cause of cardiovascular mortality (44). Vascular endothelial cells, macrophages, and vascular smooth muscle cells (VSMCs) are key cellular players in the initiation and progression of atherosclerosis.

Endothelial dysfunction increases vascular permeability, facilitating LDL infiltration into the subendothelial space, where ROS oxidize LDL to form pro-atherogenic ox-LDL (45). Macrophages phagocytose ox-LDL and differentiate into foam cells (46), meanwhile, ox-LDL and inflammatory factors trigger medial VSMC migration to the intima, with subsequent foam cell death forming a necrotic core surrounded by a VSMC- and collagen-rich fibrous cap (45, 47).

Ferroptosis, an iron-dependent regulated cell death driven by lipid peroxidation, is closely implicated in atherosclerosis, with distinct ferroptosis susceptibility across plaque subregions. The plaque shoulder, an area of high mechanical stress and active inflammation, and the necrotic core, the hub of lipid and iron accumulation, show far higher ferroptosis activity than the stable fibrous cap (48). Lesion macrophages and foam cells exhibit intense lipid peroxidation alongside sharply reduced GPX4 (the key lipid peroxide-detoxifying enzyme) and xCT (SLC7A11, the light chain of System Xc^−^) expression (49, 50). Additionally, macrophages in atherosclerotic lesions show significant accumulation of ferritin (the intracellular iron storage protein) and transferrin receptor 1 (TfR1, which mediates iron uptake), suggesting dysregulated iron homeostasis that favors ferroptosis (51). Furthermore, studies have confirmed that the progression of atherosclerosis is linked to ferroptosis in vascular endothelial cells: treatment with the ferroptosis inhibitor Ferrostatin-1 (Fer-1) reduces iron accumulation and lipid peroxidation in endothelial cells, upregulates SLC7A11 and GPX4 expression, inhibits ferroptosis in aortic endothelial cells, and ultimately alleviates atherosclerotic damage (52). Similarly, use of the intracellular iron chelator desferrioxamine (DFO) reduces cellular iron burden and inhibits iron-dependent ROS generation, thereby delaying the development of atherosclerotic lesions (53). Collectively, this evidence strongly supports a close association between ferroptosis and the development of atherosclerosis.

### Vascular calcification

Vascular calcification (VC)—the pathological deposition of calcium phosphate crystals in the vascular wall—is an independent predictor of cardiovascular risk and is closely linked to the pathogenesis of hypertension. Experimental evidence has directly linked iron metabolism to VC: intravenous iron injection in rats induces systemic iron overload, leading to arterial iron deposition and lipid peroxidation (a key feature of ferroptosis), which ultimately triggers calcification in the arterial media (50).

Osteogenic transformation of VSMCs is the central event in VC, and ferroptosis plays a pivotal role in driving this process. The lipid component POVPC [1-palmitoyl-2-(5'-oxo-valeroyl)-sn-glycero-3-phosphocholine] —abundant in atherosclerotic plaques—induces oxidative stress in VSMCs, which in turn elevates ROS levels, disrupts mitochondrial function, and triggers VSMC ferroptosis (54). Additionally, elevated serum phosphate levels (a common risk factor for VC) significantly upregulate lipocalin-2 (LCN2) expression in VSMCs. LCN2 binds to nuclear receptor coactivator 4 (NCOA4)—a selective autophagic receptor for ferritin—accelerating the degradation of ferritin heavy chain 1 (FTH1) and releasing free iron (Fe²+) into the cytoplasm (55). Intracellular free Fe²+ then generates ROS via the Fenton reaction, leading to lipid peroxidation and ferroptosis.

Ferroptosis further promotes VC by two key mechanisms: (1) it upregulates the expression of osteogenic markers (e.g., RUNX2, BMP2) in VSMCs (56), driving their differentiation into osteoblast-like cells; (2) lipid peroxide products [e.g., 4-hydroxynonenal (4-HNE), malondialdehyde (MDA)] stimulate the release of pro-calcific factors (e.g., TGF-*β*1), which accelerate hydroxyapatite deposition in the vascular wall, ultimately resulting in VC (57).

Currently, multiple potential strategies have been developed for preventing and treating VC by targeting ferroptosis (57). Ferroptosis inhibitors (e.g., Ferrostatin-1) and iron chelators (e.g., deferoxamine) can be directly administered to reduce the accumulation of free Fe²+ and lipid peroxidation (58). Metformin, a classic hypoglycemic agent, enhances the antioxidant capacity of VSMCs by activating the Nrf2 pathway, while fisetin, oleoylethanolamide, and other compounds alleviate calcification through regulating ferroptosis-related molecules (59). Additionally, restoring iron metabolic and redox homeostasis can be achieved by inhibiting PARP1-mediated POLG PARylation, downregulating the activity of HDAC9 and PKD (60), or activating the FOSL1/PHGDH-p53/SLC7A11 axis (38). Blocking LCN2-mediated NCOA4/FTH1-dependent iron release reduces the initiating substrates of ferroptosis at the source, thereby inhibiting VSMC ferroptosis and osteogenic transdifferentiation (55).

### Heart failure

Ferroptosis is increasingly recognized as a key contributor to the pathophysiology of heart failure, particularly through its effects on cardiomyocytes. Genetic evidence supports this link: cardiomyocyte-specific knockout of the ferritin heavy chain (Fth)—a critical subunit of the ferritin complex—reduces the cell's iron storage capacity, leading to elevated intracellular free Fe²+ levels. Free Fe²+ generates ROS via the Fenton reaction; simultaneously, Fth deficiency downregulates the cystine/glutamate transporter SLC7A11, limiting cystine uptake and impairing glutathione (GSH) biosynthesis (GSH is an essential cofactor for GPX4). This dual disruption of redox balance triggers an oxidative stress cascade that ultimately leads to left ventricular hypertrophy, myocardial fibrosis, systolic dysfunction, and overt heart failure (61). Other study have demonstrated that nuclear receptor coactivator 4 (NCOA4) can specifically bind to the heavy chain of ferritin (FtH), initiating ferritinophagy and releasing free Fe²+, thereby expanding the labile iron pool (LIP) (62). Additionally, lipocalin-2 (LCN2) can indirectly induce cellular apoptosis by promoting myocardial iron accumulation (63). These multiple mechanisms synergize to ultimately trigger left ventricular hypertrophy, myocardial fibrosis, and systolic dysfunction, driving the onset and progression of overt heart failure (64).

Pharmacological and molecular interventions targeting ferroptosis have shown therapeutic potential in heart failure. For example, estradiol (E2) improves cardiac function in mice subjected to transverse aortic constriction (TAC)—a model of pressure overload-induced heart failure—by upregulating dihydroorotate dehydrogenase (DHODH). DHODH promotes the reduction of coenzyme Q (CoQ) to its antioxidant form (CoQH2) and inhibits myocardial iron deposition, thereby suppressing ferroptosis (65). In diabetic cardiomyopathy-associated heart failure, the LGR6-STAT3-Pgc1*α* signaling pathway mitigates cardiomyocyte ferroptosis by preserving mitochondrial integrity; activation of this pathway improves cardiac function and alleviates heart failure in diabetic mice (66). These findings highlight the critical role of ferroptosis in heart failure progression and validate it as a potential therapeutic target.

### Ischemia-reperfusion injury

Myocardial ischemia-reperfusion injury (MIRI) is a common complication during blood flow restoration after acute coronary syndrome (e.g., myocardial infarction), and ferroptosis is a major mediator of this damage.

The molecular mechanisms linking IRI to ferroptosis have been elucidated: during myocardial ischemia, double-stranded DNA (dsDNA) accumulates in the cytoplasm, activating the cyclic GMP-AMP synthase (cGAS)-stimulator of interferon genes (STING) signaling pathway. Activated STING directly promotes the autophagic degradation of GPX4; reduced GPX4 levels lead to a marked increase in the lipid peroxidation product MDA, exacerbating ferroptosis. Notably, specific deletion of STING in cardiomyocytes reduces oxidative stress and ferroptosis, thereby alleviating myocardial IRI (67). During the reperfusion phase, reoxygenation stress elicits a massive generation of ROS via the Fenton reaction, which acts in synergy with iron metabolic dysregulation.

Cellular iron transport and metabolic regulation play a pivotal role in this process. Upregulated iron uptake mediated by the cardiac-specific transferrin receptor 2*β* (TfR2*β*) and divalent metal transporter 1 (DMT1), coupled with suppressed iron efflux via ferroportin (FPN), ultimately leads to free Fe²+ overload in cardiomyocytes (68), thereby providing a core substrate for lipid peroxidation. In contrast, redox homeostasis elicits the dual effects of ROS/nitrogen species to activate the RISK/SAFE pathways (69). The synergistic dysregulation of iron metabolism and redox balance thus represents a key regulatory target for cardioprotection in IRI. As a central signaling hub for ferroptosis in IRI, mitochondria undergo membrane potential depolarization and cristae structural damage accompanied by mitochondrial Fe²+ release, which not only exacerbates ROS production and triggers ferroptosis via Drp1-mediated excessive fission, but also participates in the crosstalk regulation of other regulated cell death pathways including necrosis and apoptosis, thereby forming a synergistic activation network of multiple cell death pathways in IRI (70, 71).

Pharmacological inhibition of ferroptosis also mitigates myocardial IRI. For instance, dexmedetomidine—an *α*2-adrenergic agonist—markedly upregulates the expression of Nrf2 (the master antioxidant transcription factor), SLC7A11, and GPX4 in cardiomyocytes (39, 72, 73). This enhancement of antioxidant capacity alleviates myocardial infarction symptoms and reduces iron accumulation and lipid peroxidation induced by reoxygenation (high oxygen exposure) in heart cells.

### Arrhythmias

Atrial fibrillation (AF)—the most common sustained cardiac arrhythmia—is closely associated with atrial fibrosis, and emerging evidence links fibrosis to ferroptosis. Mitochondrial sirtuin 3 (SIRT3)—a deacetylase that regulates mitochondrial redox balance—plays a key role in this process: SIRT3 deficiency leads to elevated acetylation of p53 (74), which suppresses GPX4 expression and promotes lipid peroxidation. In SIRT3-knockout mouse hearts, 4-HNE levels are significantly increased, GPX4 expression is downregulated, and cardiomyocytes undergo ferroptosis. Ferroptosis-derived lipid peroxides (e.g., 4-HNE) further stimulate TGF-*β*1 expression, which promotes fibroblast-to-myofibroblast transformation [marked by *α*-smooth muscle actin (*α*-SMA) positivity] and excessive extracellular matrix (ECM) secretion, leading to atrial fibrosis— a key structural substrate for AF (75).

Non-coding RNAs also regulate ferroptosis in AF. The long non-coding RNA TSNA-5008A is significantly upregulated in the myocardium of AF patients. Overexpression of TSNA-5008A promotes cardiomyocyte ferroptosis, whereas its silencing inhibits ferroptosis by regulating SLC7A11 expression and attenuates myocardial fibrosis (76). Importantly, the pro-ferroptotic effects of TSNA-5008A contribute to the formation of an arrhythmogenic substrate (76)—including calcium handling abnormalities and oxidative stress—which increases the susceptibility to AF.

## Spatial metabolomics: A powerful tool for deciphering ferroptosis mechanisms in cardiovascular diseases

### Technical principles and advantages of spatial metabolomics

Spatial metabolomics operates on the core principle of MSI, where metabolites are desorbed and ionized directly from tissue sections, with their spatial coordinates preserved to map distribution patterns. Three primary techniques dominate its application in ferroptosis-CVD research: desorption electrospray ionization MSI (DESI-MSI), gas cluster ion beam secondary ion MSI (GCIB-SIMS), and matrix-assisted laser desorption/ionization MSI (MALDI-MSI). DESI-MSI uses charged electrospray droplets to desorb metabolites from fresh-frozen tissues, achieving 50–100 μm resolution; it excels at profiling lipids and requires minimal sample preparation, making it ideal for analyzing atherosclerotic plaques and infarcted myocardium (48). GCIB-SIMS employs high-energy gas clusters to ionize samples, reaching subcellular resolution (50–100 nm) that enables detection of low-abundance metabolites like GSH and colocalization of ferroptotic signals with organelles (e.g., mitochondria, where lipid peroxidation initiates), critical for understanding subcellular ferroptosis regulation (48). MALDI-MSI uses a matrix to enhance metabolite ionization, covering both polar (e.g., iron chelators, acyl-CoA) and nonpolar metabolites, and can be integrated with immunohistochemistry to link metabolite profiles to specific cell types (e.g., macrophages in atherosclerotic lesions), strengthening connections between ferroptotic metabolism and cellular pathology (77). The key advantages of spatial metabolomics over bulk methods include: identifying “ferroptotic hotspots” (e.g., infarct border zones in MIRI), mapping crosstalk between iron metabolism, redox balance, and lipid peroxidation in region-specific CVD microenvironments, and validating *in situ* engagement of ferroptosis targets by therapeutic agents (e.g., ferrostatin-1 reducing PEox levels) (78–80).

### Current research for spatial metabolomics in deciphering ferroptosis mechanisms in cardiovascular diseases

#### Atherosclerosis

Atherosclerotic plaques exhibit marked spatial heterogeneity in ferroptotic activity, with lipid-rich necrotic cores (LRNCs) and subendothelial layers showing distinct metabolic signatures. Li et al. (48) applied DESI-MSI to human carotid plaques from 12 patients, identifying 32 differentially abundant lipids across four pathological stages. Lipid-rich regions (LRRs) are enriched in sphingomyelin (SMs), ceramide (Cer 34:1), and phosphatidylethanolamine (PE 34:2), while collagen-rich regions (CRRs) are abundant in phosphatidylcholine (PCs) and lysophosphatidylcholine (Lyso PC 20:3). Oxidized derivatives of PE and PC serve as key substrates for lipid peroxidation, whose region-specific distribution suggests spatial heterogeneity in ferroptosis-associated lipid metabolism within atherosclerotic plaques. Pathway enrichment analysis reveals that the sphingolipid signaling pathway and necroptosis pathway are significantly enriched in LRRs, whereas glycerophospholipid metabolism and ether lipid metabolism pathways are activated in CRRs. Given that sphingolipid dysregulation modulates oxidative stress and aberrant metabolism of glycerophospholipids is a core feature of ferroptosis, these region-specific pathways may indirectly regulate ferroptosis in the plaque microenvironment by modulating lipid peroxidation or oxidative stress responses (48).

#### Myocardial ischemia-reperfusion injury (MIRI)

MIRI triggers ferroptosis predominantly in reperfused myocardial regions, but spatial metabolomics has uncovered unexpected layer-specific variability. Tan et al. (81) used MALDI-MSI to analyze cardiac tissues of MIRI mice, identifying its core metabolic features. At 6 h of reperfusion, the myocardium formed metabolic partitions of ischemic injury, border and remote normal zones. Long-chain phospholipids (PA, PE, PC, PS) were enriched in the remote zone, downregulated in the border zone and markedly reduced in the injury zone, with specific PC subtypes (PC 22:6/18:0, 18:0/18:0) decreasing persistently in the injury zone. At 24 and 7 d of reperfusion, the sphingolipid metabolic pathway was significantly enriched, showing substantial GalCer accumulation and Gal₂Cer reduction in the injury and border zones, alongside marked upregulation of GLA (the enzyme catalyzing Gal₂Cer hydrolysis to GalCer) in the border zone (81). These metabolic changes align well with ferroptosis mechanisms, providing indirect yet critical metabolomic evidence for ferroptosis involved in myocardial I/R injury. Aberrant glycerophospholipid metabolism provides core lipid substrates for ferroptosis, and sphingolipid metabolic disorder modulates cellular ferroptosis susceptibility by regulating oxidative stress and membrane stability. Future studies should verify the pathological association between ferroptosis and myocardial I/R injury by detecting ferroptosis markers (e.g., MDA, 4-HNE, GPX4 activity) and cellular iron levels, as well as the effects of ferroptosis inhibitor interventions.

#### Cardiomyopathies

Diabetic cardiomyopathy (DCM) is characterized by myocardial metabolic reprogramming and cardiac dysfunction as core pathological features, with its lipid metabolism disorders, oxidative stress, and iron homeostasis-related pathological microenvironment mediating ferroptosis and contributing to disease progression. Using AFADESI-MSI and MALDI-MSI techniques, Liu et al. detected that the hearts of DCM rats exhibit a reduction in PUFA (e.g., FA 18:2, 20:4), and enrichment of phospholipids (e.g., PC 34:2, PC 36:2) in fibrotic lesions. Additionally, significantly elevated levels of GSH, Na+, and K+ were observed in DCM rat hearts, indicating oxidative stress and ionic homeostasis disruption (82). In the DCM state, enhanced oxidative stress induces preferential peroxidation of PUFA-containing phospholipids, triggering ferroptosis-related lipid damage. Although GSH is upregulated compensatorily to counteract injury, sustained oxidative stress exhausts antioxidant reserves, leading to decreased GPX4 activity and impaired clearance of lipid peroxides, ultimately increasing cardiomyocyte susceptibility to ferroptosis.

### Breakthroughs and limitations for ferroptosis-cardiovascular disease research

**Figure 5:**
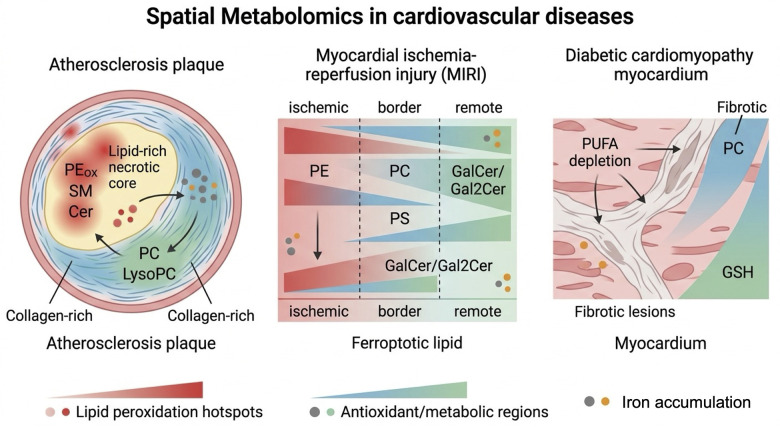
Spatial metabolomics in deciphering ferroptosis mechanisms in cardiovascular diseases.

Spatial metabolomics has addressed longstanding gaps in ferroptosis-CVD research, driving four key breakthroughs. First, it validates mechanistic links between iron metabolism, redox balance, and lipid peroxidation in CVD-specific microenvironments. Second, it identifies prognostic spatial biomarkers for CVD. Third, it accelerates therapeutic development by enabling *in situ* validation of ferroptosis inhibitors. Collectively, these breakthroughs position spatial metabolomics as an indispensable tool for advancing ferroptosis-based research and therapy in CVDs (Figure 5).

Despite metabolomics' progress in dissecting ferroptosis-related metabolic changes, spatial metabolomics remains underutilized in CVD-focused ferroptosis research, leaving critical gaps (Table 2). Current studies rely mostly on bulk tissue analysis, which averages metabolite profiles across heterogeneous cell populations and fails to capture spatial heterogeneity of ferroptosis-associated metabolites, such as lipid peroxide accumulation in myocardial ischemia-reperfusion injury's infarct border zone, thereby obscuring metabolite-ferroptosis spatial correlations. While spatial metabolomics could identify region-specific biomarkers and guide precision therapy, such applications are nascent, existing studies lack integration with other omics, and platform-related high costs and technical complexity restrict adoption, delaying translation to ferroptosis-targeted interventions.

**Table 2:** Integrative metabolomic landscape of ferroptosis in cardiovascular diseases.

Metabolic axis	Key metabolites biomarkers	Pathophysiological mechanism	Representative cardiovascular model	Key studies (year)
Iron metabolism	Fe²+, ferritin, transferrin, FTH1	Iron overload drives ROS accumulation and lipid peroxidation, initiating ferroptotic death	Myocardial ischemia–reperfusion injury (I/R); diabetic cardiomyopathy	Tan et al. (81)*; Zhao et al. (66)
Lipid peroxidation	PUFA–PE, ALOX15, ACSL4, MBOAT7	PUFA-phospholipid oxidation forms cytotoxic lipid hydroperoxides, amplifying ferroptotic stress	Diabetic Cardiomyopath; ischemic myocardium	Liu et al. (82)*; Wang et al. (73)
Glutathione metabolism	Cys, GSH, GPX4, SLC7A11	Antioxidant defense controlling peroxide detoxification; depletion enhances ferroptosis	Ischemia–reperfusion injury; HFpEF models	Yao et al*.* (39); Su et al*.* (74)
Mitochondrial energ*y* axis	CoQ10, DHODH, NADPH, AMPK	Energy imbalance and redox collapse promote mitochondrial ferroptosis	Heart failure; post-ischemic remodeling	Wang et al*.* (65)
Amino acid/lipid crosstalk	Neu5Ac, LPC/LPE ratios, FFAs	SLC3A2 degradation and ether-lipid remodeling modulate ferroptosis susceptibility	Atherosclerosis, vascular endothelium	Xiang et al*.* (52); Su et al*.* (50)
Spatial metabolomic features	Peroxidized PE hotspots, 4-HNE, Fe^2+^ distribution	Subepicardial ferroptosis zones; mitochondrial iron aggregation	I/R myocardium; progressive HF	Tan L et al*.* (81)*
Therapeutic modulation	AMPK agonists, DHODH activators, CoQ10 supplementation	Restores redox homeostasis and suppresses ferroptosis-dependent remodeling	Preclinical cardiomyopathy & ischemia models	Wu et al*.* (84); Wang et al*.* (65)

*Studies of spatial metabolomics.

## Critical issues and limitations

Firstly, while the core drivers of ferroptosis have been extensively characterized, the dynamic crosstalk among distinct signaling pathways and the inter-organellar communication mechanisms between subcellular compartments remain poorly defined and unsystematically delineated, respectively. Compounding these limitations, current research predominantly focuses on isolated molecular mechanisms rather than adopting a comprehensive systems biology perspective. To address these gaps, exploring interactions between ferroptosis and the cardiovascular immune microenvironment or gut microbiota-derived metabolites may emerge as a novel and impactful research direction. Furthermore, integrating artificial intelligence (AI) with multi-omics datasets to construct dynamic network models of ferroptosis could uncover previously unrecognized regulatory nodes.

Secondly, current ferroptosis diagnosis relies on indirect indicators (e.g., lipid peroxidation products MDA and 4-HNE) or histological features (e.g., mitochondrial morphological alterations), with a lack of highly specific, non-invasive biomarkers. Existing detection technologies also suffer from inadequate resolution and sensitivity for dynamically monitoring ferroptosis progression *in vivo*. Therefore, developing novel biomarkers based on multi-omics approaches or advanced molecular imaging techniques represents a critical future direction.

Thirdly, current research predominantly relies on preclinical models and *in vitro* assays, with clinical validation lagging considerably. Gene-knockout murine models and drug-induced pathological systems fail to fully recapitulate the complexity of human CVDs. Meanwhile, *in vitro* cell studies often rely on supraphysiological drug concentrations or extreme oxidative stress conditions, which raises valid concerns about the physiological relevance of their findings. Though iron chelators and lipid peroxidation inhibitors show preclinical efficacy, their human safety, effectiveness and long-term risks are underexplored, and even multi-target agents like metformin lack clinical data for specific ferroptosis modulation. Moreover, ferroptosis-targeted interventions carry off-target risks—systemic iron chelators may exacerbate anemia or disrupt iron-dependent processes [12], and the potential interference of lipid peroxidation inhibition with normal signaling demands further systematic evaluation.

Lastly, the contribution of ferroptosis may vary substantially across distinct CVD subtypes, yet current studies often generalize findings using single pathological models (e.g., myocardial ischemia-reperfusion injury). For instance, ferroptosis in atherosclerosis primarily involves vascular smooth muscle cells, whereas cardiomyocyte ferroptosis dominates in heart failure. However, the similarities and disparities in their underlying regulatory mechanisms remain unclear [76]. Moreover, the impact of individual variations (e.g., sex, genetic background, comorbidities) on ferroptosis susceptibility has scarcely been investigated—creating a critical gap in the context of precision medicine (83).

## Conclusion

**Figure 6:**
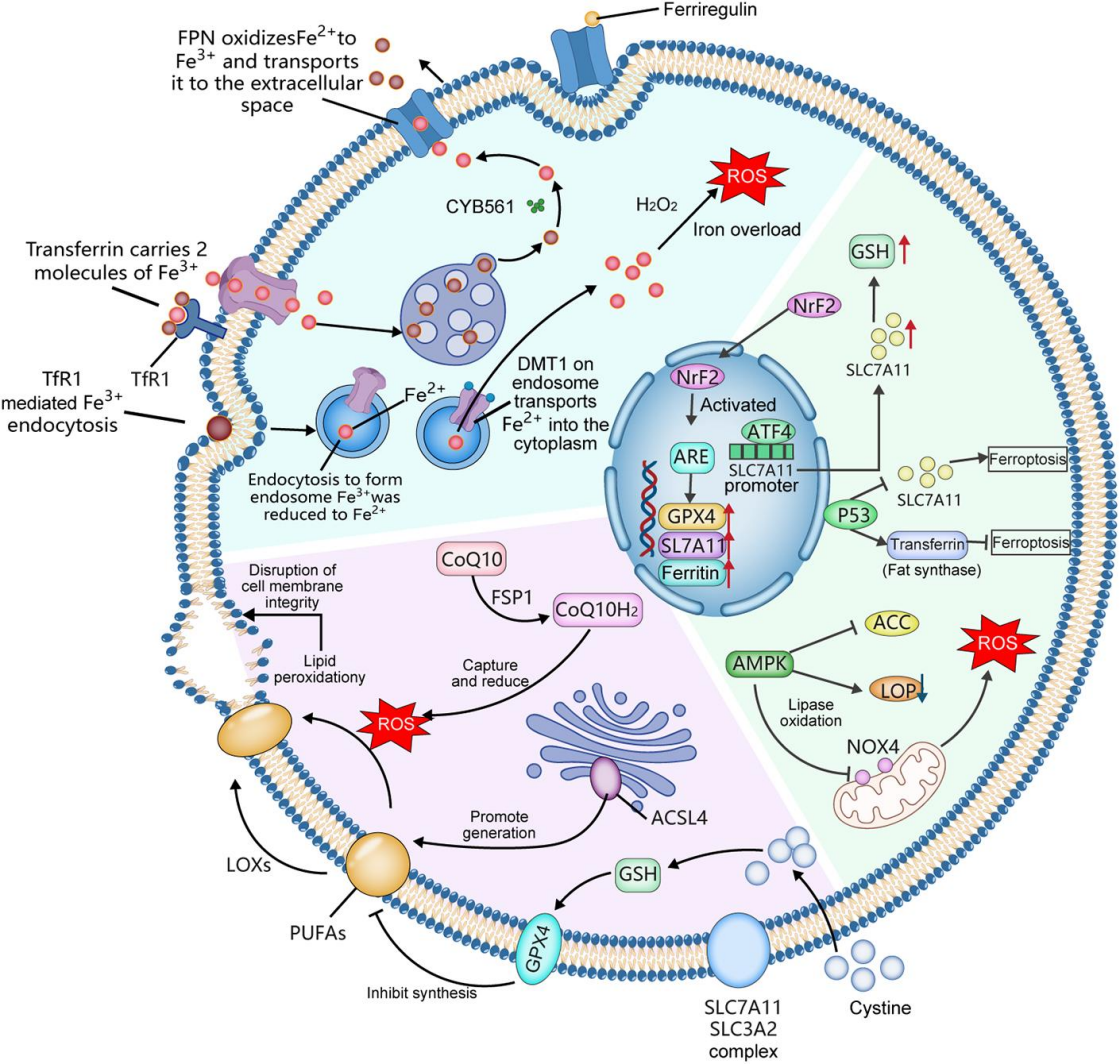
The key molecular mechanisms related to ferroptosis within cells. ACSL4, acyl-CoA synthetase long-chain family member 4; GPX4, Glutathione peroxidase 4; GSH, glutathione; Nrf2, nuclear factor erythroid 2-related factor 2; AMPK, AMP-activated protein kinase; ROS, reactive oxygen species; TfR1, transferrin receptor 1; FPN, ferroportin; PUFAs, polyunsaturated fatty acids; LOXs, lipoxygenases.

As an iron-dependent, lipid peroxidation-driven form of regulated cell death, ferroptosis has emerged as a pivotal mechanism in the pathological progression of CVDs. This review systematically summarizes the roles of iron metabolism, lipid peroxidation, and core regulatory molecules, clarifies the regulatory network of subcellular organelles, and highlights ferroptosis' central involvement in conditions such as atherosclerosis and heart failure (Figure 6). Notably, spatial metabolomics provides a critical tool for deciphering region-specific ferroptotic mechanisms. The review confirms ferroptosis as a promising therapeutic target for CVDs, with targeted interventions showing potential for clinical translation. Future research should focus on exploring crosstalk between multiple pathways, developing specific biomarkers, advancing clinical validation, and integrating multi-omics with artificial intelligence to optimize precision therapeutic strategies, thereby offering new avenues for CVD prevention and treatment.
